# Assessment of Medication Adherence Using Mobile Applications in Chronic Obstructive Pulmonary Disease: A Scoping Review

**DOI:** 10.3390/ijerph21101265

**Published:** 2024-09-24

**Authors:** Belen Machado, Pamela Quimbaya, Rosa-Helena Bustos, Diego Jaimes, Katherinne Cortes, Daniela Vargas, Laura Perdomo

**Affiliations:** 1 Clinical Pharmacology Department, Faculty of Medicine, Universidad de La Sabana, Campus del Puente del Común, Chía 250001, Cundinamarca, Colombia; gloriamano@unisabana.edu.co (B.M.); pamelagoqu@unisabana.edu.co (P.Q.); 2 Evidence-Based Therapeutics Group, Department of Clinical Pharmacology, Faculty of Medicine, Universidad de La Sabana and Clínica Universidad de La Sabana, Campus del Puente del Común, Chía 250001, Cundinamarca, Colombia; diegojf@unisabana.edu.co; 3 Faculty of Medicine, Universidad de La Sabana, Chía 250001, Cundinamarca, Colombia; kt.cortesp@gmail.com (K.C.); danielavaro@unisabana.edu.co (D.V.); lauraperrod@unisabana.edu.co (L.P.)

**Keywords:** medication adherence, pharmacological adherence, chronic obstructive pulmonary disease, mHealth, mobile applications

## Abstract

Chronic obstructive pulmonary disease (COPD) is a condition that significantly impacts both patients and healthcare systems. The management of COPD involves various pharmacological intervention strategies, and addressing the issue of low adherence to these strategies has become a subject of significant interest. In response to this concern, there has been a shift toward utilizing telemedicine and mobile applications. The primary objective of this scoping review is to delineate the usage of mobile applications to enhance medication adherence in adult patients with COPD. This study involved a search of databases such as Medline, Google Scholar, Cochrane, and ClinicalTrial.gov, focusing on the literature published in English and Spanish over the last decade. The selected studies assessed interventions involving mobile applications (mobile apps) designed to improve medication adherence. Four digital aids were identified and available on online platforms, mobile apps, or both: m-PAC, myCOPD, Wellinks mHealth, and Propeller Health. Propeller Health, in particular, is an app that directly measures medication adherence through electronic medication monitors attached to participants’ inhalers. Opening the app was associated with higher odds of using control medications compared to participants who did not open the app. The findings suggest that these digital interventions serve as valuable tools to enhance patient adherence to treatment. Future research should focus on evaluating the effectiveness of different digital devices, such as digital inhalers and mobile applications, that directly measure medication adherence.

## 1. Introduction

Chronic obstructive pulmonary disease (COPD) has been identified as a major contributor to the global burden of disease [1]. Chronic diseases are conditions that last for one year or more, which may or may not limit daily life activities and typically require ongoing medical attention. Among adults with chronic conditions, it is estimated that 30% to 50% of medications are not taken as prescribed [2]. According to the World Health Organization (WHO), adherence to medication regimens can have a greater impact on patient outcomes than the specific treatment itself [2], and nonadherence poses a significant economic burden on healthcare systems.

Poor adherence to treatment plays a crucial role in the progression of this condition. It is quite common among patients with COPD, ranging from 20% to 60%, resulting in exacerbation of symptoms, frequent hospitalizations, and poor quality of life [3]. One of the most prevalent concerns is the inadequate use of medication, with up to 49.4% not adhering to nebulized treatments as prescribed, 31% using ineffective techniques for inhaler dosing, and over 50% overusing medications during respiratory difficulty [4]. In COPD, the most commonly identified risk factor for involuntary nonadherence is the complexity of medication regimens, including both oral and inhaled therapies and polypharmacy, which is more frequent in patients over 65 years of age with comorbidities [5,6,7].

There are multiple modes of drug administration for COPD, including conventional inhalers, dry powder inhalers, capsules for dry powder inhalation, and nebulizers. The existence of these diverse and often complex methods, particularly in an aging population, may hinder patients’ ability or willingness to adhere to treatment regimens [8].

According to The EMERGE Guidelines (Empowering Medication Adherence and Improving Clinical Outcomes), recommendations are aimed at standardizing and improving medication adherence assessment in clinical research. Adherence is defined as the extent to which a patient’s behavior aligns with the prescribed medical treatment. It involves three key phases: (i) Initiation: When the patient takes the first dose of the medication. (ii) Implementation: The degree to which a patient follows the prescribed dosing schedule. (iii) Discontinuation: When the patient stops taking the medication before the prescribed course is completed [9]. Different strategies have been identified for the assessment of medication adherence in COPD and have been classified as subjective or objective, and these, in turn, can be classified as direct or indirect [10].

There is increasing interest in leveraging mobile applications to enhance healthcare outcomes and improve adherence, with promising results seen in other diseases, such as asthma [11] and cardiovascular disease [12], which may be applicable to COPD.

Mobile health (mHealth) is a term used to describe medical practice and health assistance supported by informatics and mobile devices such as tablets, mobile phones, etc. A meta-analysis by Fen Yang et al. [13] evaluated the effectiveness of mobile health applications as interventions to reduce hospital admissions and length of hospitalization. Additionally, a recent Cochrane review assessed the effectiveness of interventions using mobile and computer technologies compared to face-to-face, digital, or printed interventions aimed at facilitating self-management among people with COPD. The review found that individuals who used innovative technologies showed significant improvements in self-management, quality of life, and physical activity [14]. However, significant clinical heterogeneity was noted in the trials included in these systematic reviews. As such, it is necessary to evaluate the available tools and mobile applications on a case-by-case basis through a scoping review to accurately assess the evidence.

Given the essential role of technology, especially mobile devices and web applications, in modern life, these innovations offer a promising avenue for improving therapeutic adherence. However, due to the limited availability of high-quality evidence, the substantial heterogeneity in the literature, potential conflicts of interest related to the economic gains from new technologies, and the scarcity of evidence demonstrating real clinical outcomes from these interventions, we propose a scoping review. This review serves as an initial step toward conducting new trials in this field to determine the true cost-effectiveness of these interventions. The main objective of this scoping review is to characterize the use of mobile applications to improve medication adherence in adult patients with COPD and to identify potential areas for future research and intervention.

## 2. Materials and Methods

To address the research question, a scoping review was conducted following the methodological guidelines proposed by Arksey and O’Malley [15], along with recommendations from Levac et al. and the Joanna Briggs Institute [16,17]. The reporting of results adhered to the published in 2018 [18]. The concepts of persistence and adherence, which are critical for achieving optimal treatment outcomes, were considered. According to ESPACOMP, adherence refers to the extent to which a patient correctly follows the prescribed medication regimen, including taking the correct dose at the appropriate times. Persistence is defined as the duration of time from initiation to the discontinuation of therapy [9]. In this study, which focuses on a chronic disease where the therapeutic goal is long-term control, persistence may take precedence because the long-term outcome depends on maintaining treatment over an extended period. However, adherence to therapy is a prerequisite for achieving persistence, making it essential to assess adherence.

### 2.1. Selection Criteria

Eligible studies for this review included interventions using mobile applications, online platforms, or electronic and wearable devices aimed at improving medication adherence in patients aged 18 or older diagnosed with COPD by spirometry. Patients at any stage of COPD severity were considered. Primary observational and experimental studies published in English and Spanish were selected, including case reports, systematic/narrative/scope reviews, and meta-analyses. Studies reporting outcomes related to medication adherence using any measurement method were included (e.g., electronic monitoring, structured or semi-structured questionnaires). Interventions focused solely on pulmonary rehabilitation or adherence to physical exercise were excluded. Additionally, conference abstracts, posters without full text, book chapters, editorials, or letters to the editor were excluded based on study type.

The PRISMA-ScR statement for scoping reviews was utilized [18] as a checklist by the two authors who conducted the search. The evaluation process commenced with the title, followed by the abstract. Subsequently, all selected papers underwent thorough reading by both authors, with specific attention given to the protocol, registration, data processing, and information sources as outlined in the PRISMA-ScR statement. The results and discussion sections were carefully assessed. Each article underwent a PRISMA-ScR checklist to evaluate its quality.

### 2.2. Search Strategy

The associated MeSH terms used were “Pulmonary Disease, Chronic Obstructive,” “health” OR “mobile app,” and “medication adherence.” Following an exhaustive search, additional words, synonyms, and exclusion operators were considered (Table S1). We conducted a comprehensive literature search using electronic databases, including Cochrane Database of Systematic Reviews, Medline, Lilacs, and Google Scholar from inception to February 2023. Two independent reviewers (Machado B and Cortes K) conducted the initial screening of titles and abstracts based on the predefined inclusion and exclusion criteria. Discrepancies between the reviewers were resolved through discussion, and a third reviewer (Bustos RH) was consulted if consensus could not be reached. Data extraction was performed by the two reviewers using a standardized data extraction form in Excel (Microsoft. Excel, 2016, Redmond, WA, USA).

The data were synthesized using an Excel table collaboratively created by both authors, including information up to February 2023, when the search was performed. The information includes details on study design, main objectives, sample size, clinical stages of COPD, and interventions conducted to assess adherence.

## 3. Results

**Figure 1:**
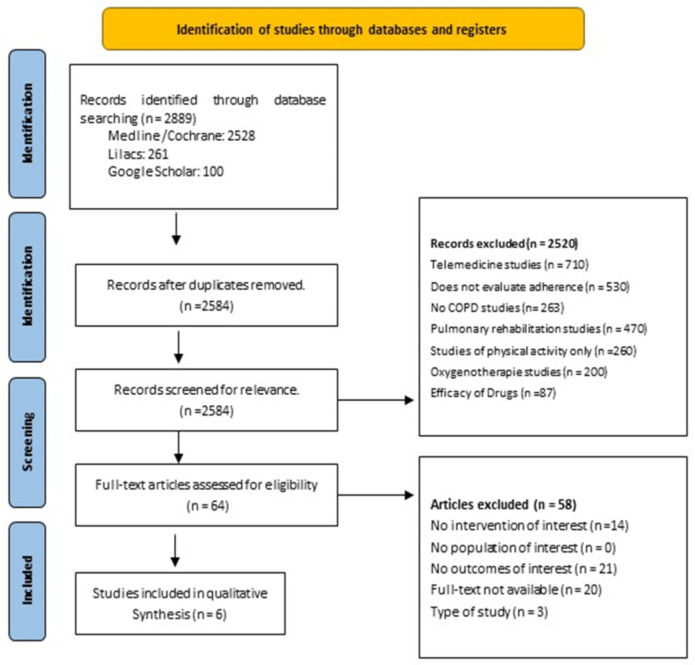
Flow chart with the paper selected.

The number of articles identified from the database search was as follows: Medline/Cochrane: 2528; Lilacs: 261; and Google Scholar: 100. After screening the titles and abstracts, 61 articles were selected for full-text review, and finally, six papers were included (Figure 1). The articles included in the review provided sufficient descriptions of their study methods to extract essential information for this review. The characteristics of the studies are highlighted in Table 1. Regarding the study types, two were clinical trials, three were observational pilot studies, two were prospective studies, and one was retrospective, along with a narrative review. These studies were conducted in different countries: the United States (n = 2), the United Kingdom (n = 1), Belgium (n = 1), the Netherlands (n = 1), and Australia (n = 1).

Regarding the interventions, two of the studies combined a mobile application with an additional sensor for their inhaler, transferring data to the app via Bluetooth [19]. Another study provided a mobile application and a study kit that included a spirometer, pulse oximeter, and nebulizer [20]. Additionally, another study combined a mobile application with a device to measure physical activity [21]. Two studies included an online application to assist COPD patients through education, self-monitoring, and self-management functions.

**Table 1:** Summary of included studies.

Name	How Was Medication Adherence Measured?	Study Design	Study Period	Sample Size	Average Years (SD)	Clinical Stage of COPD	Pharmacological Adherence Results	Refs.
Mobile App								
m-PAC (App)	Patients were asked a weekly question about changes to their medication via the smartphone app.	Randomized controlled clinical trial	2019–2021	42	58 ± 18	Mild, moderate, severe, and very severe	The overall percentage of answers to the question about medication was 72%, and a change of medication was reported in 7.6% of the questions answered.	[19]
N.S.	N.A.	Narrative review	N.S.	N.A.	N.A.	N.S.	Mobile apps connected to digital inhalers offer several advantages: They provide patients with real-time reminders to take their medication, which helps to improve adherence; they can also track medication use and offer inhalation technique visualization, allowing patients to identify and correct any errors in their inhalation technique.	[22]
Online tool								
myCOPD	The study measured drug adherence by analyzing data recorded in the myCOPD application. Participants were able to enter their medication use into the app, allowing for follow-up and adherence monitoring.	Open clinical trial	2018–2019	60	66.1 ± 7.1	Mild, moderate, and severe	The study did not directly measure the relationship between outcomes and medication adherence; however, it did find that increased use of the app was associated with clinically significant benefits on the COPD Assessment Test (CAT) score.	[20]
Online tool + app								
Wellinks mHealth	Medication adherence was measured by manually entering the daily medication into the application, which contained a personalized list of medications prescribed to each COPD patient.	Prospective observational pilot study	Jan–May 2021	19	76.9	Mild, moderate, severe, and very severe	Medication use entries decreased from 7.8 times per week to 3.7 times per week, indicating a 52.3% reduction in drug adherence.	[23]
Propeller Health	Medication adherence was measured using EMMs that were attached to participants’ inhalers. Adherence was calculated by dividing the number of actions recorded by the EMM by the prescribed number of actions reported by the participant during enrollment, multiplied by 100 per day.	Retrospective study (analysis	2018–2019	2309	60.9 ± 8.3	N.S.	In participants with COPD (n = 663), opening the app on a given day was also associated with greater odds of using controller medications compared with participants who did not open the app (OR 1.61 (1.49, 1.75); *p* < 0.001).	[24]
Electronic health								
Self-management intervention (without tradename)	Medication adherence and inhalation technique were monitored using a complementary inhalation sensor (Respiro^®^), which was compatible with the Ellipta^®^ inhaler.	Prospective (descriptive) pilot study	2018–2019	11	66.8 ± 2.9	Mild, moderate	Short inhalations (<1.25 s) were the most common inhalation error (79.6%).	[21]

N.S.: not specified; N.A.: not applied; m-PAC: project-tailored smartphone; COPD: chronic obstructive pulmonary disease; COPD Assessment Test (CAT) score; CAT: COPD Assessment Test; EMMs: Electronic Medication Monitors mobile applications available focused on pharmacological adherence in patients with COPD and measurement of medication adherence m-PAC.

### 3.1. m-PAC (Mobile Physical Activity Coaching)

m-PAC is a smartphone application developed explicitly for the physical activity training program for patients with COPD [19]. The application includes functions integrated with a device (Fitbit) and a weekly medication question. This approach aims to detect exacerbations or other adverse events in real time by tracking changes in medication through the smartphone application. Medication adherence was indirectly evaluated through medication changes reported by the patients in response to the weekly question posed by the app and subsequent conversations with the coach. The results showed that the weekly medication question formulated through the smartphone application could effectively detect exacerbations in patients with COPD. The response rate to the medication question was 72%, and medication changes were reported in 7.6% of cases. Of the 18 exacerbations recorded during the 6-month follow-up period (among 42 patients included), ten moderate exacerbations were detected through the medication question in the training app. However, it was observed that adherence to the medication question decreased over time, which could pose challenges for detecting exacerbations or adverse events.

### 3.2. MyCOPD

MyCOPD is an online application designed to assist patients with COPD through education, self-monitoring, and self-management features [20]. The healthcare team registered study participants and then activated the application at their convenience through an email link. The application allows users to track their symptoms, medication usage, and inhalation techniques. It also offers educational resources on COPD treatment, such as videos and articles. Users can schedule medication and appointment reminders and access a community forum to share support and information. The study evaluated the feasibility and potential effectiveness of using the myCOPD application in patients with mild to moderate chronic obstructive pulmonary disease (COPD). Specific details on how pharmacological adherence was measured in the study are not provided in the available information; however, it can be inferred that adherence was evaluated based on the frequency and consistency of medication input recorded by the participants in the app. The study showed good engagement with the myCOPD application, with sustained usage patterns for up to 90 days. Although the study did not directly measure the relationship between outcomes and pharmacological compliance, it found that increased app usage was associated with clinically significant benefits in the COPD Assessment Test (CAT) score, a measure of disease activity and symptomatic burden in patients with COPD. Signals of improvement in inhalation technique and disease activity score were also observed, suggesting the potential effectiveness of the myCOPD application.

### 3.3. eHealth (Self-Management Intervention)

The mobile application offers various modules and features to support self-management in patients with multiple chronic diseases [21]. Patients can access the application through a tablet or smartphone with an internet connection. The application includes a digital symptom diary where patients can record their symptoms related to COPD, heart failure, and other comorbidities. In addition, patients receive an additional sensor for their inhaler, which transfers data to the application via Bluetooth. The application indicates if inhalations are performed correctly and provides audiovisual reminders for scheduled medication doses. Overall, the application aims to assist patients in managing their chronic diseases by providing tools for symptom monitoring, education, self-treatment guidance, and medication adherence support.

Medication adherence was measured by calculating adherence levels to inhaled medication, dividing the number of times patients used their inhaler by the number of times they should have used it according to their doctor’s prescription, and evaluating adherence to the inhalation technique. The study used an additional inhaler sensor called Respiro^®^ (by Amiko Digital Health Limited, London, UK), and the inhaler used in the study was the Ellipta^®^ inhaler (GlaxoSmithKline BV, Brentford, Middlesex, London, UK). Although adherence to daily symptom tracking and use of inhaled medication was high, commitment to recommended actions and inhalation technique was inadequate. The results showed that patients had high adherence to inhaled medication, with an adherence rate of 98.4% (752 out of 764 days). The range of inhalations performed among patients was from 96.3% to 100%. However, the percentage of correctly performed inhalations varied among and within patients, with only 51.9% being performed correctly. The most common inhalation error was inhaling for too short a time (<1.25 s).

### 3.4. Wellinks mHealth App

This application is an integrated and multimodal digital health solution for patients with COPD [23], which allows them to register and monitor various aspects of their condition. Patients use their smartphones to download the app, which securely collects and stores data related to their treatment and respiratory status. Additionally, patients receive a study kit that includes three devices—the Flyp nebulizer, the MIR Smart One spirometer, and the Nonin pulse oximeter—which are connected to the app via Bluetooth. Patients are required to perform spirometry and pulse oximetry measurements at least once a week with the provided devices, and these measurements are automatically recorded in the app.

Patients also manually input their daily medication and symptoms into the app. The app includes a personalized list of prescribed medications for each COPD patient, and patients can mark when they take each dose. The app generates monthly reports summarizing medication adherence, Forced Expiratory Volume (FEV)1 and SpO2 measurements, and symptoms, which are emailed to the doctor. The study assessed the feasibility and patient satisfaction with the app’s usage. Medication adherence was measured by manually inputting daily medication data into the Wellinks mHealth App. The app included a personalized list of prescribed medications for each COPD patient, and patients could mark when they took each dose. Medication usage was entered into the app nine times per week, and symptoms were entered 1.2 times per week. Over time, medication usage entries were reduced from 7.8 times per week to 3.7 times per week (a 52.3% reduction) over eight weeks.

The platform demonstrated a high level of patient engagement and satisfaction and the ability to collect and store treatment and respiratory status data. The study results suggest that the Wellinks platform can improve patient engagement and outcomes in COPD management, ultimately reducing related events and expenses.

### 3.5. Propeller Health

Propeller Health is a smartphone application that includes an Electronic Medication Monitor (EMM), which connects to a compatible inhaler to record the date and time of use passively [24]. The EMM usage data are wirelessly transferred via Bluetooth. The application offers various features and content related to asthma and Chronic Obstructive Pulmonary Disease (COPD) treatment, including relevant guidelines, medication usage and trends, and medication reminders based on scheduling through the EMM and smartphone app. Users are also prompted to complete an Asthma Control Test (ACT) or COPD Assessment Test (CAT) in the app at the beginning and monthly after that to assess disease control and burden. The article aimed to explore the relationship between app engagement and medication adherence using objective data collected from an actual sample of adults with asthma and COPD enrolled in a digital health platform.

Pharmacological adherence was measured using data from the participant’s controller inhaler connected to the EMM. The EMM recorded the date and time of each inhaler actuation, enabling adherence calculation. Adherence was determined by dividing the number of recorded actions by the prescribed number of daily activities and multiplying by 100. The burden of COPD was assessed using the COPD Assessment Test (CAT), an 8-item questionnaire. Participants rated their symptoms on a scale of 1 to 5, and total scores were classified as lower burden (<20) or higher burden (≥20). Over 90 days, unadjusted mean medication adherence was higher among participants with COPD than with asthma: 62% (32%) versus 45% (32%), respectively. In participants with COPD (n = 663), opening the app on a given day was also associated with higher odds of using control medications compared to participants who did not open the app (OR 1.61 (1.49, 1.75); *p* < 0.001). The study found that participants who engaged with the app, based on app usage data, were more likely to achieve their medication adherence goals.

### 3.6. Digital Inhalers and Their Impact

According to Bosnic-Anticevich et al., “adherence” and “inhalation technique” are widely used when discussing inhalers by patients with asthma and COPD. This narrative review aimed to assess the impact of digital inhalers on clinical and economic outcomes and identify knowledge and practice gaps in this field [22].

Digital inhalers for COPD incorporate built-in sensors and digital technology to passively record and provide information on various parameters related to inhaler use. An example of a digital inhaler for COPD is the Digihaler^®^ for albuterol (Teva Pharmaceuticals, LLC, Tel Aviv, Israel), which was the first FDA-approved inhaler with integrated sensors capable of recording and providing information on maximum inspiratory flow (MIF), time to MIF, inhalation volume and duration, as well as adherence data. Another example is the Respiro^®^ RS01X device (Amiko, Milan, Italy), which also incorporates sensors that provide information on each critical movement during inhaler actuation. Additionally, there are other intelligent inhalers available or undergoing evaluation in clinical trials for COPD treatment, such as the recently approved Enerzair^®^ Breezhaler^®^ (Novartis Pharmaceuticals, Basel, Switzerland.), which can be paired with the Propeller^®^ mobile app to help physicians and patients monitor and record inhaler device management.

**Figure 2:**
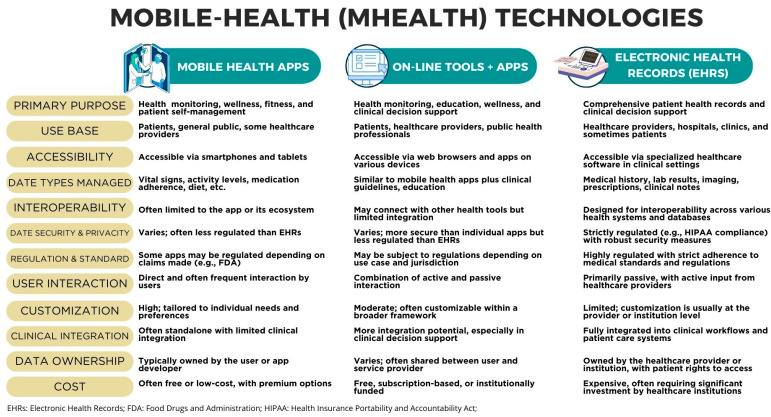
Comparison of mobile health technologies described in the results of this study. Comparisons were presented according to categories inherent to the technology, such as accessibility, technology, and interoperability. Categories of cost, customization, and clinical integration are also mentioned [25,26,27,28,29].

Patients with respiratory diseases like asthma and COPD often need correct inhalation techniques, resulting in suboptimal medication administration and poor disease control. Digital inhalers offer the opportunity to address this issue by providing objective measurements and guidance to patients, allowing them to correct their technique and optimize medication use. Furthermore, they offer the opportunity to apply an identification and risk reduction approach by monitoring patient behaviors and identifying patterns that may precede asthma and COPD exacerbations. These digital devices can track parameters such as inhaler adherence, inhalation technique, and symptomatic patterns, providing valuable data that can be used to identify patients at risk of exacerbations and intervene early to prevent them. Figure 2 shows the comparison and characteristics of each of the m-health technologies selected and discussed in this article.

## 4. Discussion

In this scoping review, an overview of the technological aids that could improve medication adherence in COPD patients is given; five tools were found, two available on the web and two in mobile applications. One combines the use of an application and a digital platform. Among the online media is MyCOPD, which, although it does not directly measure medication adherence, does have the option of messages where you receive notifications to inform the doctor if any change in medication or frequency has been made; in the study, it was found that greater use of the application was associated with improvement in inhalation technique and the score of disease activity, suggesting a possible efficacy of the application.

The technology could be vital to improving this indicator of adherence to health interventions, especially medication adherence. In 2017, Sobnath et al. reviewed mobile applications, COPD management systems, and the literature to identify the characteristics of a mobile application for this pathology, finding overwhelmingly educational apps. Very few related to treatment, with no description of medication adherence, concluding that the apps identified in the market are limited in terms of functionalities. Very few emphasize the needs of COPD patients with comorbidities [30].

The Wellinks and m-PAC applications both present virtual solutions for COPD management, utilizing mobile apps and online platforms. However, their respective adherence monitoring results reveal contrasting outcomes. In the case of Wellinks, despite its comprehensive approach combining a mobile app and an online platform, the study demonstrated a decrease in the average number of medication entries over the 8-week period. While the online platform may indirectly assess medication adherence, it lacks the ability to measure proper inhalation techniques. Moreover, external variables such as internet connectivity or platform accessibility remain unaccounted for, introducing potential confounding factors that demand careful consideration in interpreting the findings.

Conversely, the m-PAC application, designed for assessing physical activity in COPD patients and incorporating a weekly medication-related question, also exhibited a decline in medication adherence over time. Similar to Wellinks, the diminishing compliance with medication-related inquiries suggests a commonality in the challenges faced by these virtual solutions in sustaining user engagement for medication monitoring.

In contrast, the Propeller Health program stands out as a physician-recommended solution, providing a more comprehensive approach to COPD management. This program integrates an inhaler sensor, a mobile app compatible with both Android and Apple devices, or an online portal, along with personalized assistance. Notably, Propeller Health distinguishes itself by incorporating evidence-based asthma and COPD content, along with direct feedback on medication use. Unlike the previous applications, Propeller Health assesses medication adherence as a primary objective, utilizing the inhaler sensor to directly capture controller inhaler use data. This method enables a retrospective analysis, demonstrating how a smartphone app dedicated to asthma and COPD management can significantly impact and improve medication adherence.

Some studies have assessed medication adherence using scales such as the Morisky Medication Adherence Scale (MMAS-4). A study using this scale demonstrated that overall adherence (OA) to inhaled COPD medication was optimal with 66.3% of participants; factors such as educational level, comorbidities (such as peptic ulcer and history of anemia), and depressive symptoms were negatively associated with adherence. The study highlighted the importance of addressing the inhaler technique, given that adherence measurement is performed non-objectively, and identified common adherence problems, such as forgetting to use medication and intentionally reducing the dose when feeling well [31]. The inhalation technique is crucial. Our study found that digital inhalers are an opportunity provided by this type of device, being the first that can provide objective information on medication use. Considering that poor adherence is associated with inhalation techniques, healthcare professionals can identify changes in patient behavior that may indicate an increased risk of exacerbations. In 2016, Killane et al. compared exacerbation prediction models using inhaler medication adherence data sources, specifically dose counter and electronic monitoring, which are significantly higher with the dose counter, ultimately proving that these data highlight the need to identify optimal metrics to develop a predictive model [32].

Furthermore, when compared to indirect adherence metrics such as self-reported adherence as surveys, they could be more reliable, mainly by providing reliable information on whether doses were inhaled or discharged or real-time electronic medication monitoring such as calls or personalized SMS reminders. However, these can be personalized and must be more comprehensive to address the complex challenge of intentional nonadherence. Intentional nonadherence is when patients deliberately choose not to follow their medication regimen. Strategies focused on unintentional nonadherence, such as reminders, may need to address the underlying reasons for intentional nonadherence effectively. A study by Vasbinder et al. found that sending personalized SMS reminders to children with asthma improved adherence to inhaled corticosteroids (ICS) but did not show improvements in disease control, quality-adjusted life years (QALYs), or exacerbation rates [33].

Few studies focus on mobile health’s effectiveness as a tool to optimize therapeutic adherence. A systematic review was conducted in 2022 investigating the quality of mobile apps in patients with chronic conditions or morbidity (defined as two or more chronic conditions) and their potential to promote lifestyle changes; however, medication adherence was not evaluated [14]. In that study, 60 mobile apps were included, of which 35 (58%) were available in both the app store and Google Play. The selected apps mainly advised changes in physical activity and diet for patients with one or more of the following conditions: knee or hip osteoarthritis, cardiovascular disease, type 2 diabetes mellitus, COPD, and depression. It can be noted that despite the growing interest in mobile health interventions to enhance therapeutic adherence, the existing literature reveals a notable gap in studies specifically evaluating medication adherence within the realm of mobile apps. A systematic review conducted in 2022 focused on the quality of mobile apps for patients with chronic conditions, yet it did not include an assessment of medication adherence.

In a systematic review, they evaluated the effectiveness of mobile apps in people with COPD for self-management; however, the study did not precisely measure medication adherence. The outcomes measured in the survey included the frequency of COPD exacerbations that resulted in the clinical intervention (hospitalization or community treatment), physical function, quality of life (physical activity, dyspnea, self-efficacy, fatigue, anxiety, and depression. The study showed no statistically significant effects of mobile apps on measures of physical function or quality of life in people with COPD [34], which means that despite the comprehensive evaluation, the findings revealed no statistically significant effects of mobile apps on measures of physical function or quality of life in individuals with COPD.

While it is commonly perceived by the general public that older individuals may struggle with using mobile applications and technology, this notion is flawed. Multiple studies have demonstrated that older individuals can learn and effectively use technology, challenging the misconception. Embracing this perspective is crucial, especially when considering the potential of technology to improve health outcomes [35]

Technological innovation is already enabling many patients with chronic disease to take greater control of their disease management and achieve better disease outcomes; it is essential to continue to generate evidence in this area; regularization of these tools and adequate support strategies for real-life implementation are required. Only then will it be possible to use the devices’ data to provide personalized disease management.

## 5. Limitations

This scoping review provides new information about mobile or online applications and medication adherence in patients with COPD. The strengths of this scoping review include a comprehensive search and review of the literature, which helps to avoid selection bias. Additionally, two researchers were involved to ensure rigor in the article selection process, and a third party resolved conflicts. However, it is essential to note that out of the six selected studies, three are pilot studies with small sample sizes. While this scoping review does not aim to establish a measure of association, it is advisable to consider other studies beyond pilot studies to obtain more robust findings for future research. Only studies in two languages were included, which could lead to the exclusion of important literature from other languages. Further research is needed to develop new theoretical models comprehensively addressing medication adherence.

## 6. Conclusions

As a result of all these digital interventions, we can determine that their use is an additional tool to help patients improve treatment adherence and become increasingly personalized. Using mobile applications is straightforward and intuitive. However, it may be limited to older adults or people with disabilities. Additionally, applications that indirectly measure adherence through surveys or questions over time tend to need help with the issue of users ceasing to complete them, making it challenging to measure compliance in this manner accurately. The use of digital inhalers linked to mobile applications stands out, as it has been found that these measure adherence in terms of inhaler usage frequency and the correct inhalation technique. This form of direct and objective adherence measurement provides an opportunity to apply an identification and risk reduction approach by tracking patient behaviors, identifying patterns preceding exacerbations, and intervening early to prevent them. In conclusion, addressing the current research gap on medication adherence within mobile health interventions is imperative to unlock the full potential of these tools in optimizing therapeutic outcomes for individuals managing multiple chronic conditions.

## 7. Future Research

Digital inhalers and mobile applications are crucial in treating respiratory diseases, as they provide accurate and objective information about medication usage, improve therapeutic compliance, and foster patient engagement. Mobile applications can facilitate communication between patients and healthcare professionals, allowing doctors to remotely monitor adherence and inhalation techniques. This information can guide therapeutic decisions and enable personalized interventions. The significance of digital inhalers and mobile applications lies in their ability to empower patients to manage their respiratory conditions. In the future, it will be essential to assess the effectiveness of digital tools combining a digital inhaler with a mobile application to evaluate medication adherence and inhalation technique, thereby increasing patient involvement in their care. This scoping review lays a solid foundation for future studies and the development of theoretical models that contribute to a better understanding of medication adherence in patients with COPD and its relationship with available mobile and online applications.

## Data Availability

The data are contained in this article.

## Supplementary Materials

The following supporting information can be downloaded at https://www.mdpi.com/article/10.3390/ijerph21101265/s1. Table S1: Search strategy.
