# Supplementary material for: Assessment of Medication Adherence Using Mobile Applications in Chronic Obstructive Pulmonary Disease: A Scoping Review
Source: Int J Environ Res Public Health. 2024 Sep 24;21(10):1265. doi: 10.3390/ijerph21101265 (PMC11506935; doi:10.3390/ijerph21101265)
Supplement: Supplementary file 1 [file ijerph-21-01265-s001.zip › ijerph-3141821-supplementary.pdf]

Table S1. Search Strategy.

Data bases	**Cochrane Database of Systematic Reviews**<br>**Cochrane Central Register of Controlled Trials**
Platform	Ovid
Filtres	Date: Feb 2012/Feb 2023
Strategy	1 exp COPD/ or pulmonary disease, chronic obstructive/ or bronchitis, chronic/ or pulmonary emphysema/ or copd.mp. or "chronic obstructive pulmonary disease".mp. [mp=ti, ab, tx, ct, kw, ot, fx, sh, hw, bt, nm, kf, ox, px, rx, ui, sy]<br>2 (mhealth or app or mobile or application).mp.<br>3 exp Telemedicine/ 46154<br>4 exp cell phone/ or smartphone/ or text messaging/<br>5 2 or 3 or 4<br>6 1 and 5
Data bases	**Medline**
Platform	Ovid
Filtres	Date : Feb 2012/Feb 2023
Strategy	1 exp COPD/ or pulmonary disease, chronic obstructive/ or bronchitis, chronic/ or pulmonary emphysema/ or copd.mp. or "chronic obstructive pulmonary disease".mp. [mp=ti, ab, tx, ct, kw, ot, fx, sh, hw, bt, nm, kf, ox, px, rx, ui, sy]<br>2 (mhealth or app or mobile or application).mp.<br>3 exp Telemedicine/ 46154<br>4 exp cell phone/ or smartphone/ or text messaging/<br>5 2 or 3 or 4<br>6 1 and 5
Data bases	**Lilacs**
Platform	Bireme
Filtres	Date : Feb 2012/Feb 2023
Strategy	((mh:(bronquitis cronica)) OR (mh:(enfermedad pulmonar obstructiva cronica)) OR (mh:(enfisema pulmonar)) OR (coad OR epoc OR evoc OR enfermedad obstructiva cronica de las vias aéreas OR enfermedad pulmonar cronica obstructiva OR enfermedad del pulmon cronica obstructiva OR neumopatia obstructiva crónica OR obstruccion cronica del flujo aereo OR obstruccion del flujo aereo cronica)) AND (((mh:(telemedicina)) OR (smartphone) OR (dispositivos moviles) OR (telefono celular)) OR (celulares OR lineas moviles OR apps OR aplicaciones moviles OR esalud OR msalud OR usalud OR salud movil OR salud digital OR salud electronica OR cibersalud))
Data bases	**Google Scholar**

Platform	Google Académico
Filtres	Date : Feb 2012/Feb 2023
Strategy	(COPD OR chronic obstructive pulmonary disease) AND (mhealth OR mobile OR app OR telemedicine OR smartphone)
Data bases	**Medline**
Platform	Pubmed
Filtres	Date : Feb 2012/Feb 2023
Strategy	(((((((Chronic Obstructive Pulmonary Disease[MeSH Terms]) OR (Chronic Airflow Obstruction)) OR (Chronic Bronchitis)) OR (Pulmonary Emphysema)) OR (Chronic Obstructive Airway Disease)) OR (Chronic Obstructive Lung Disease)) OR (COPD)) AND ((((((((((((Mobile Applications[MeSH Terms]) OR (telemedicine[MeSH Terms])) OR (Smartphone[MeSH Terms])) OR (Mobile Apps)) OR (Portable Electronic Applications)) OR (Portable Electronic Apps)) OR (Portable Software Applications)) OR (Portable Software Apps)) OR (Smartphone Apps)) OR (Mobile Health)) OR (Telehealth)) OR (eHealth)) OR (mHealth))) AND ((((((((((((((((((((Treatment Adherence and Compliance[MeSH Terms]) OR (medication adherence[MeSH Terms])) OR (patient compliance[MeSH Terms])) OR (Drug Adherence)) OR (Drug Compliance)) OR (Medication Compliance)) OR (Medication Non-Adherence)) OR (Medication Non-Compliance)) OR (Medication Nonadherence)) OR (Medication Noncompliance)) OR (Medication Persistence)) OR (Therapeutic Adherence)) OR (Non-Adherent Patient)) OR (Patient Adherence)) OR (Patient Cooperation)) OR (Patient Non-Adherence)) OR (Patient Non-Compliance)) OR (Patient Nonadherence)) OR (Patient Noncompliance)) OR (Therapeutic Compliance)) OR (Treatment Compliance))
